# Sexual Hallucinations in Schizophrenia Spectrum Disorders and Their Relation With Childhood Trauma

**DOI:** 10.3389/fpsyt.2018.00193

**Published:** 2018-05-09

**Authors:** Jan Dirk Blom, Esmeralda Mangoenkarso

**Affiliations:** ^1^Parnassia Psychiatric Institute, The Hague, Netherlands; ^2^Faculty of Social and Behavioural Sciences, Leiden University, Leiden, Netherlands; ^3^Department of Psychiatry, University of Groningen, Groningen, Netherlands

**Keywords:** erotic hallucination, genital hallucination, incubus phenomenon, multimodal hallucination, sexual trauma

## Abstract

**Background:** Sexual hallucinations are probably the most neglected types of hallucination, even in psychiatric settings. They are often multimodal in nature, and their prevalence rate is unknown. For other types of hallucination, notably auditory hallucinations, childhood trauma is an important risk factor. However, whether this also applies to sexual hallucinations is unexplored.

**Objective:** To establish the prevalence rate of sexual hallucinations in a clinical sample of patients diagnosed with a schizophrenia spectrum disorder, to describe their phenomenological characteristics, and to estimate their relationship with childhood trauma.

**Methods:** After screening 778 patients diagnosed with a schizophrenia spectrum disorder, 42 were considered eligible for inclusion by their treating physician or psychiatrist. Thirty of these patients were interviewed to assess the presence of sexual hallucinations, using a tailor-made questionnaire and the short form of the Childhood Trauma Questionnaire.

**Results:** Of the 30 patients interviewed, 13 reported sexual hallucinations, yielding a 1-year prevalence rate of 0.017 in this clinical sample. Of the hallucinating patients, 46.2% reported multimodal hallucinations, with involvement of up to five sensory modalities. All patients who experienced sexual hallucinations reported a history of childhood trauma, of which 76.9% involved sexual trauma (OR 8.7). In addition, 61.5% of the patients reported high levels of distress.

**Conclusion:** In patients diagnosed with a schizophrenia spectrum disorder, sexual hallucinations warrant appropriate medical attention. They are not as rare as traditionally thought, and their relationship with childhood trauma is overwhelming. Therefore, we recommend that clinical attention be paid to the psychotic *and* traumatic symptoms of these patients, as well as to the somatic conditions that may underlie them. For clinical and research purposes, we propose a classification of sexual hallucinations in accordance with the sensory modalities involved. As sexual hallucinations are also experienced in the context of temporal lobe epilepsy, narcolepsy, persistent genital arousal disorder, intoxications and other somatic conditions, further research in transdiagnostic populations seems warranted. In line with the current practice of providing trauma-focused treatment for trauma-related auditory hallucinations, we recommend that future studies explore the effectiveness of this type of treatment for sexual hallucinations.

## Introduction

Sexual hallucinations are probably the most neglected types of hallucination, even in psychiatric settings. This may be due to the fact that the sexual modality falls outside the five basic sensory modalities (i.e., olfaction, taste, vision, audition, and touch) and that they are, therefore, hardly on the radar of health professionals and clinical researchers, especially when they are not familiar with more extensive classifications of the sensory modalities, which may list up to 14 of them (1). Another reason may be that patients suffering from sexual hallucinations are likely to feel embarrassed to talk about these phenomena. Alternatively, health professionals may expect their patients to be embarrassed and, hence, refrain from asking about them. Moreover, when other types of hallucination provide sufficient reason to initiate pharmacological treatment (as in the presence of distressing auditory hallucinations) the need to explore the presence of sexual hallucinations may appear superfluous to clinicians. Thus, there appear to be various reasons why these phenomena tend to go unnoticed and are considered very rare. However, we believe this is more a case of their being unreported or unexplored as few systematic studies on this topic have been conducted, implying that sound prevalence rates of sexual hallucinations are still lacking.

Clinical practice indicates that these types of hallucination can be extremely burdening. Based on our experience at a secluded nursing ward for patients diagnosed with schizophrenia spectrum disorders, we know that patients sometimes report the taste of sperm in their mouths, describe the bewildering sensation of being changed into a person of the opposite sex, or experience random orgasmic feelings which seriously disrupt their ability to function, especially in social situations. Others may be plagued by hallucinated sex odors, or display suicidal and/or aggressive behavior because of the hallucinated sensation of being sexually harassed; in the case of a young woman without a psychiatric history, this ended in actual suicide within weeks after she had been admitted to our hospital. Although not all sexual hallucinations have such a dramatic outcome, the latter example may serve to underline the importance of the need to pay appropriate medical attention to these underreported and virtually unexplored phenomena.

In the last decade, childhood trauma has become firmly established as a risk factor for hallucinations of the verbal auditory type (i.e., hearing voices), notably in the context of schizophrenia spectrum disorders (2–5). To investigate whether this might also hold true for sexual hallucinations, we screened all patients newly admitted to two nursing wards of our psychiatric hospital to establish the presence of sexual hallucinations and childhood trauma. From this clinical sample we describe 13 cases and provide data on the prevalence and frequency of sexual hallucinations in this group, the phenomenological characteristics of the hallucinations, the levels of ensuing distress, and associations with childhood (sexual) trauma. Some recommendations are also made for further research and clinical practice.

## Materials and methods

### Subjects

From July 2012 through June 2014, we requested all psychiatrists and psychiatric residents in the service of two nursing wards for patients diagnosed with a schizophrenia spectrum disorder at Parnassia Psychiatric Institute (The Hague), to refer any patients whom they might suspect *at that moment* of experiencing sexual hallucinations (i.e., present state). These hallucinations were operationalized as (i) tactile hallucinations involving touch to erogenic zones such as the penis, vagina, buttocks, or breasts; (ii) somatic hallucinations involving penetration, misplaced feelings of sexual lust or random orgasmic sensations; (iii) visual and/or verbal auditory hallucinations with an explicit sexual content; and (iv) hallucinated tastes and smells of a sexual nature. Patients were considered eligible for inclusion when they experienced one or more of these hallucinations. Other inclusion criteria were age 18–65 years, and a DSM-IV diagnosis of schizophrenia or a related psychotic disorder, as established by the treating psychiatrist. Exclusion criteria were a diagnosis of substance-induced psychotic disorder or psychotic disorder due to a somatic condition, an IQ <70, and insufficient mastery of the Dutch or English language. After oral and written explanation of the study, written informed consent was obtained from all participating patients.

### Instruments and procedures

All interviews were carried out by a psychiatric resident (E.M.) under supervision of a clinical psychiatrist (J.D.B.). During the interviews, and on the basis of the patients' medical files, demographic data were collected regarding age, gender, marital status, level of education, and clinical diagnosis. In addition, two questionnaires were administered. The first one was a tailor-made, semi-structured questionnaire specifically designed for the present purpose (Data Sheet 1), which contained 27 questions about the presence of sexual hallucinations and delusions during the past 2 weeks, the nature of the delusions (if present), the phenomenological characteristics of the hallucinations (if present), and ensuing levels of distress. All items allowed for binary answers (“present” or “absent”), as well as for free-text responses to ensure optimal capture of phenomenological particulars. The second questionnaire was the short form of the Childhood Trauma Questionnaire [CTQ-SF; (6)], which was employed to explore possible relations between reported sexual hallucinations and any childhood adversities, if present. The CTQ-SF is a validated self-report questionnaire [also for psychotic disorders, see (7)] which contains 25 questions to assess the presence and severity of five different types of childhood trauma, i.e., physical abuse, sexual abuse, emotional abuse, emotional neglect, and physical neglect. Each trauma category is assessed with the aid of five statements for which participants select a level of frequency, i.e., never true, rarely true, sometimes true, often true or very often true. These responses are then coded on a 5-point Likert scale. Total CTQ-SF scores range from 25 to 125, with each individual abuse subscale ranging from 5 to 25, and higher scores indicating more severe types of abuse.

### Data analyses

Data were analyzed using SPSS version 23.0 and Microsoft Excel. Descriptive statistics were calculated for each variable of interest. Chi quadrate tests and Fisher's exact test were used to assess the association between sexual hallucination and childhood trauma. Logistic regression was used to analyse these associations, and correct for different confounders between childhood traumatic experiences and sexual hallucinations. Statistical significance was defined as *p* < 0.05.

## Results

### Demographic data

During the 2-year inclusion phase, a total number of 2,285 admissions took place at the two nursing wards. With almost half of the patients being admitted more than once (sometimes as often as 10 times a year), the total number of individual patients was 778. Of these, 42 (5.4%) were considered eligible for participation by their treating physicians. Eight of these patients refused to participate, and four were discharged before they could be included. The remaining 30 (3.9%) were interviewed. Of these, 19 (63.3%) were male, and 14 (46.7%) were non-Caucasian. The mean age of the participants was 39 (SD 12; range of 19–64) years; 17 participants (56.7%) had completed municipal high school or higher forms of education, and 10 (33.3%) were in a relationship. All patients were diagnosed with schizophrenia or a related psychotic disorder in accordance with the DSM-IV, with three of them (10%) having a comorbid diagnosis of posttraumatic stress disorder (PTSD). None of the participants had a diagnosis of substance abuse disorder, although some of them did occasionally use alcohol, cannabis or other illicit substances.

### Sexual hallucinations

**Figure 1:**
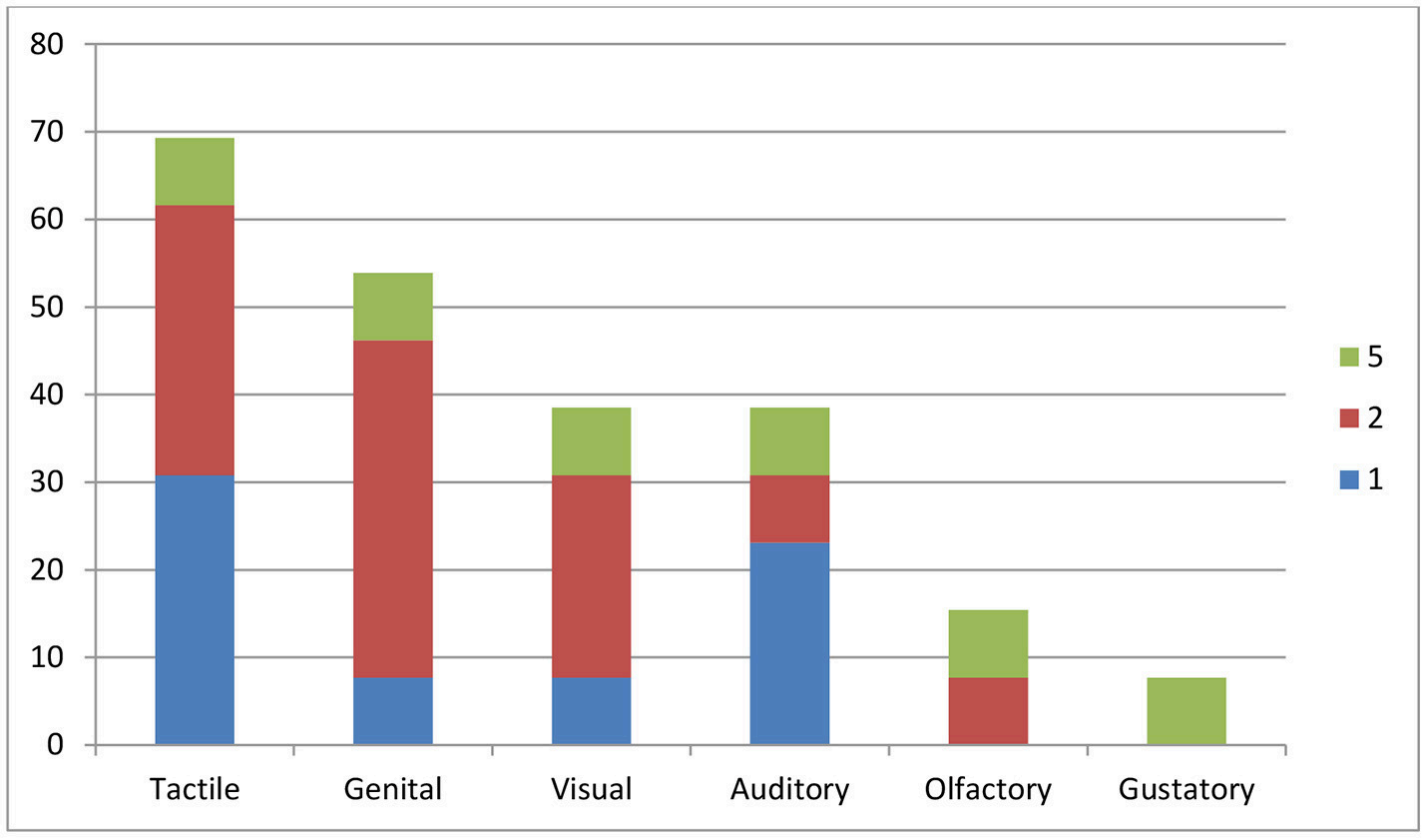
Percentage of patients experiencing **s**exual hallucinations, per sensory modality. The x axis indicates the sensory modality involved; the y axis the percentage of participants (*n* = 30) experiencing hallucinations in that particular sensory modality. Color codings indicate the presence of multimodal hallucinations (in up to five sensory modalities). As some of the participants experienced hallucinations in more than one sensory modality, the total percentage is higher than 100.

Of the 30 participants, 13 (43.3%) experienced sexual hallucinations (Table 1), of which nine (30%) experienced sexual hallucinations *and* delusions. Of the remaining 17 (56.7%), five (16.7%) experienced only sexual delusions, and 12 (40%) delusions of a different nature. Based on these data, the 1-year prevalence of sexual hallucinations in our clinical sample was 0.017. Figure 1 describes the distribution of the reported hallucinations per sensory modality. Tactile sexual hallucinations were reported most frequently, i.e., by nine of the 13 hallucinating participants (69.2%). Among them, seven (53.8%) experienced genital hallucinations, and two (15.4%) sexually charged hallucinations affecting different body parts. Both visual and verbal auditory hallucinations with an explicit sexual content were experienced by five participants (38.5 and 38.5%, respectively), whereas olfactory hallucinations involving sex odors were reported by two participants (15.4%), and gustatory ones by only one (7.7%). Remarkably, six participants (46.2%) reported sexual hallucinations in multiple sensory modalities (see color codings in Figure 1), either serially (serial multimodal hallucination) or simultaneously (compound hallucination), with the involvement of up to five sensory modalities.

**Table 1:** Overview of the 13 participants experiencing sexual hallucinations.

**Age (range in years)**	**DSM-IV diagnosis**	Type of trauma^*^	**Type of sexual hallucination**	**Reported distress**
30–35	Schizoaffective disorder	EN	Tactile (genital and other body parts), visual	None
26–30	Schizophrenia	SA	Auditory	None
46–50	Psychotic disorder NOS	EN/PN	Auditory, tactile (genital and other body parts)	Anger, helplessness, depression
46–50	Schizophrenia	EA/PA/SA	Auditory, tactile (genital and other body parts), visual, olfactory, gustatory	Anxiety
36–40	Psychotic disorder NOS	EA/PA/SA/EN/PN	Tactile (genital and other body parts), visual	Anxiety, shame, anger
60–65	Schizophrenia	EA/PA/SA/EN/PN	Auditory	None
16–20	Psychotic disorder NOS	EA/PA/SA	Tactile (genital and other body parts), olfactory	Depression, anxiety
30–35	Schizophrenia	EA/PA/SA/EN/PN	Visual	None
26–30	Schizophrenia	SA/PN	Tactile (genital)	None
20–25	Schizophrenia	EA/EN/PN	Tactile (genital), visual	Anger, anxiety
40–45	Schizophrenia	PA/SA/PN	Tactile (non-genital)	Pleasant sensations
20–25	Schizophrenia, PTSS	SA/EN	Tactile (non-genital)	Anxiety, depression
50–55	Schizophrenia	EA/SA	Auditory	Anxiety

* *EN, emotional neglect; PN, physical neglect; EA, emotional abuse; SA, sexual abuse; PA, physical abuse*.

Some examples of qualitative descriptions of the sexual hallucinations were as follows. One participant described that it felt as if her skin and genitals were being touched while there was no-one around. As a consequence, she attributed these tactile hallucinations to a ghost. In addition, she described sexually charged electrical shocks applied to the genitals, breasts, and anus. Another participant experienced fingers running over his body which he, too, attributed to a ghost. The sensation of being penetrated and sexually abused was mentioned by several patients, as were visual images of people having sexual intercourse. Two participants reported nightly visits by an intruder who climbed upon them to have sexual intercourse with them (i.e., an incubus phenomenon). In addition, one of these two participants reported continuous day-time sexual hallucinations. One participant who experienced auditory hallucinations reported a voice commanding him to visit his partner for the purpose of initiating sexual intercourse. Another participant heard a voice urging him to masturbate, while yet another one reported hearing random orgasmic sounds. As regards gustatory and olfactory hallucinations, one participant mentioned “*the smell of sperm and orgasms*,” and another one mentioned the taste of sperm.

### Distress

Of the hallucinating participants, eight (61.5%) reported high levels of distress during their hallucinatory episodes. Associated emotions were fear, anger, helplessness, depression, and shame. Only one patient (7.7%) reported pleasant emotions, and the four remaining ones (30.8%) reported no associated emotions at all.

### Trauma

Of the 30 participants, 26 (86.7%) reported having experienced a childhood trauma in one form or another. Sexual abuse was the most common variant, reported by 16 of the participants (53.3%), followed by emotional abuse (15 participants; 50%), physical neglect (14 participants; 46.7%), physical abuse (13 participants; 43.3%), and emotional neglect (12 participants; 40%). For all participants, the mean score on the CTQ-SF was 54.7 (S.D. 17.5). Men and women had comparable scores: 54.6 (S.D. 19.0) and 54.8 (S.D. 15.3), respectively. No significant differences were found between men and women on the various subscales of the childhood trauma scores.

In the subgroup of participants who experienced sexual hallucinations, all 13 reported at least one form of childhood trauma. Regarding sexual trauma, 10 of them (76.9%) replied positively vs. six (i.e., 35.3%) from the group with no sexual hallucinations (*p* = 0.024). Table 2 shows the rates for different forms of childhood trauma as reported by the hallucinating vs. the non-hallucinating patients. No significant differences were found between the two groups for any of the other forms of childhood trauma. An odds ratio (OR) of 8.7 was calculated for sexual trauma during childhood and later reports of sexual hallucinations, after correction for age, sex, and type of trauma (*p* = 0.028), meaning that the chance to experience sexual hallucinations after childhood sexual trauma was 8.7 times higher than in people without such a history.

**Table 2:** Rates for various types of childhood trauma as reported by patients experiencing sexual hallucinations, vs. those experiencing no sexual hallucinations.

	**Patients reporting sexual hallucinations (*n* = 13)**	**Patients reporting no sexual hallucinations (*n* = 17)**	***p*-value**
Mean total score on the CTQ-SF (SD)	59.1 (12.7)	51.4 (20.1)	*p* = 0.057
Trauma	100% (13)	76.5% (13)	*p* = 0.113
Physical neglect	53.8% (7)	41.2% (7)	*p* = 0.491
Physical abuse	46.2% (6)	41.2% (7)	*p* = 0.785
Emotional abuse	53.8% (7)	47.1 (8)	*p* = 0.713
Emotional neglect	53.8% (7)	29.4 (5)	*p* = 0.219
Sexual abuse	76.9% (10)	35.3 (6)	*p* = 0.024

*CTQ-SF, short form of the Childhood Trauma Questionnaire*.

## Discussion

### Prevalence

In this clinical sample of 778 acutely admitted patients diagnosed with a schizophrenia spectrum disorder, a 1-year prevalence of 0.017 was found for sexual hallucinations. Considering that some 70–80% of patients diagnosed with a schizophrenia spectrum disorder report hallucinations in general (8), this figure is indeed very low. However, very few studies are available for a direct comparison, since none of the 10 large-scale epidemiological surveys of hallucinations carried out in the general population singled out sexual hallucinations as a separate category [for an overview see (9)], and clinical surveys of sexual hallucinations are rare. Moreover, of the seven clinical surveys that we could retrieve (Table 3) most were performed during the 1960s; all of them were smaller (50–150 inclusions) and all reported substantially higher prevalence rates. Thus, even though most studies were performed in a clinical setting and among population groups comparable to our own, older prevalence rates range from 0.08 to 0.57. This may be due to methodological differences, since the older studies did not always provide case definitions, not all studies explicitly stated whether they focused on the present-state or lifetime presence of sexual hallucinations, and not all studies indicated whether or not their patients were randomly recruited. Thus, the sexual hallucinations described by Gittleson and Levine (10) and Gittleson and Dawson-Butterworth (11) are in fact genital hallucinations, defined in their study as “*genital feelings regarded by the patient as of unusual or abnormal type*,” while the highest prevalence rate reported in the literature, as established by Klaf (12), was based on a study that pooled sexual hallucinations and delusions together in a single category. As a consequence, the reported prevalence of sexual hallucinations of the latter sample cannot be accepted at face value.

**Table 3:** Prevalence rates of sexual hallucinations as reported in earlier studies.

**Study**	**Type of study, duration**	**Population (gender)**	**N**	**% Sexual hallucinations**
Klaf and Davis (35)	Retrospective, on the basis of case records	Adult clinical population, schizophrenia (males)	150	26.7% (including delusions with a sexual content and delusions of infidelity)
Klaf (12)	Retrospective, on the basis of case records	Adult clinical population, schizophrenia (females)	75	57.3% (including delusions with a sexual content)
Small et al. (36)	Prospective, duration unknown	Adult clinical population, schizophrenia (males and females)	50	8% (tactile)
Gittleson and Levine (10)	Prospective, 6 months	Adult clinical population, schizophrenia (males)	70	30% (genital)
Gittleson and Dawson-Butterworth (11)	Prospective, 3 months	Adult clinical population, schizophrenia (females)	57	44% (genital)
Lyketsos et al. (37)	Cross-sectional	Adult clinical population, schizophrenia (males and females)	113	19.5% (14.1% auditory, 5.3% visual, 3.5% bodily/tactile, 1.8% olfactory, 0.9% forced orgasms)
Thompson et al. (13)	Prospective, 6 months	Adolescent and young adult outpatient population at ultra-high risk for psychosis (males and females)	92	9.7% (6.5% auditory, 3.2% visual)

The most recent survey of sexual hallucinations, by Thompson et al. (13), probably comes closest to the present study, even though it included a different population group. After recruiting 92 patients from an Australian outpatient facility for young individuals aged 15–24 years at ultra-high risk for psychosis, Thompson and colleagues found that 6.5% reported auditory hallucinations with a sexual content and 3.2% visual hallucinations; there were no reports of tactile sexual hallucinations. Although the authors do not state whether the visual and auditory hallucinations were experienced by different individuals, the prevalence of sexual hallucinations in their outpatient sample of individuals with attenuated psychotic symptoms appears to be 0.097. This, too, is a relatively high rate compared with our findings, especially considering the much broader definition of sexual hallucinations that we used (covering seven rather than three sensory modalities) and the fact that our participants were more severely ill. As a consequence, we consider the rate that we established as very conservative.

### Historical perspective

Historically, descriptions of sexual hallucinations have been very rare. For millennia, the only exception seems to have been the incubus phenomenon, i.e., a compound hallucination, experienced during spells of sleep paralysis, involving a person, animal or metaphysical being exerting pressure on the thorax, and occasionally subjecting its victim to sexual intercourse or other types of sexual harassment (14). The condition has been known since Antiquity and, up until the nineteenth century, was attributed to metaphysical beings such as demons or fallen angels (15). Other descriptions dating back to Antiquity are those of rare instances of satyriasis and nymphomania, i.e., an insatiable desire for sexual stimulation accompanied by dysaesthesia of the genitals and unbidden orgasmic sensations (16). During the nineteenth century, Parish (17) briefly described sexual hallucinations in the context of hysteria, in cases where nitrous oxide was administered, and (referencing Boerhaave) intoxications with *Atropa belladonna* and *Datura stramonium*. Bleuler (18) merely remarks that tactile and somatic hallucinations in the context of schizophrenia often start out as genital sensations, while von Krafft-Ebing (19), in his *Psychopathia Sexualis*, describes a man who, under the influence of large quantities of Indian hemp (i.e., cannabis), reported that his body had transformed overnight into that of a woman, complete with female genitals and breasts.

From the 1940s onwards, various case reports and modest case series have been published on sexual hallucinations in the context of hypoparathyroidism (20), schizophrenia (21–24), mental retardation (25), narcolepsy (26, 27), epilepsy (28, 29), and the use of anesthetics (30, 31). In the latter category, especially nitrous oxide has become a controversial substance because of its capacity to induce sexual hallucinations, and because of the alleged misuse of that knowledge by health professionals who made themselves guilty of actual sexual harassment while blaming their victims for their ignorance of the side-effects of nitrous oxide (32). Worth a special mention is persistent genital arousal disorder (PGAD) (33), formerly known as persistent sexual arousal syndrome (PSAS) (34), which is basically a modern reconceptualization of nymphomania. While PGAD was initially associated with clitoral engorgement and clitoral priapism under the influence of trazodone and related compounds, more recent studies indicate that it may be caused by pelvic conditions such as pudendal nerve neuropathy, pelvic congestion syndrome, and Tarlov cysts (16), thereby suggesting that any unbidden orgasmic sensations experienced in this context are not necessarily hallucinations.

### Etiology and pathophysiology

This brief historical overview shows that sexual hallucinations are experienced in the context of widely varying disorders and syndromes. Their occurrence in the context of schizophrenia spectrum disorders may suggest that mesolimbic dopaminergic pathways are involved in their mediation; however, their occurrence in other conditions indicates that there may be different causal pathways. Thus, sexual hallucinations experienced after initiation or withdrawal of selective serotonin reuptake inhibitors (SSRIs) and serotonin-norepinephrine reuptake inhibitors (SNRIs) suggest the involvement of serotonergic mechanisms (38). Regarding sexual hallucinations following the administration of nitrous oxide, the exact working mechanism is unknown, although blockade of NMDA glutamate receptors is proposed as a likely candidate (39). As the incubus phenomenon is experienced most often in the context of narcolepsy, the underlying mechanism of sexual hallucinations in either condition is possibly a dissociation of sleep phases and the subsequent intrusion of dream contents (40). Based on an electroencephalographic study by Rémillard et al. (28), orgasmic hallucinations in the context of epilepsy have been linked to the limbic portion of the temporal lobe in the non-dominant hemisphere. Since the literature on PGAD suggests that local pelvic conditions may also be involved, it is safe to say that the etiopathology of sexual hallucinations is probably multifactorial in nature, and certainly requires further elucidation. An important consequence for clinical practice is that somatic conditions, such as those mentioned here, need to be ruled out, even in the presence of a psychiatric diagnosis of schizophrenia spectrum disorder.

### Trauma

As has been shown, childhood trauma (notably sexual childhood trauma) is another risk factor for sexual hallucinations. As proposed by Howes and Murray (41) in their integrated sociodevelopmental-cognitive model, its influence on the mediation of hallucinations and other psychotic symptoms may well be integrated with the factors mentioned above. In line with our findings, Thompson et al. (13) established a significant relationship between the presence of attenuated psychotic symptoms with a sexual content and prior sexual trauma, with an OR of 7.17 (*p* < 0.01). This comes close to the OR of 8.7 that we calculated in the present study, and indicates that trauma is a risk factor worthy of further exploration and medical attention. Especially since trauma-focused therapy is no longer considered a contraindication for patients diagnosed with schizophrenia spectrum disorders (42), this may have important therapeutic consequences, even for those patients who claim that sexual hallucinations do not cause them any distress (as did 38.5% of our sample). The reason why so many patients reported a lack of distress is unknown to us, but we speculate that it may be connected with the relatively high prevalence of a blunted affect in this patient group. Alternatively, patients may have enjoyed these sensations, or they may have had a calming or soothing effect on them. If so, this might well have consequences for the need to treat these symptoms, as well as for the effects to be expected.

### Classification

In the present sample, sexual hallucinations were experienced in the olfactory, gustatory, visual, auditory, somatic, and tactile modalities, with 46.2% being of a multimodal nature. This is in line with the finding that multimodal hallucinations, rather than auditory hallucinations, are the most frequent type of perceptual symptom in the context of schizophrenia spectrum disorders (1). As some studies on sexual hallucinations restrict themselves to genital hallucinations, whereas others use broader definitions that include up to seven sensory modalities (43), we propose the use of a classification that may help to clarify diagnostic procedures for both clinical and research purposes (Table 4). Because the term “genital hallucination” refers to a body part rather than to a sensory modality, it may be advisable to replace this term by either “tactile hallucination” or “somatic hallucination,” or else add adjectives to specify the type of hallucination (i.e., tactile genital hallucination, somatic genital hallucination). Likewise, we recommend to reclassify “orgasmic hallucinations” as “somatic hallucinations,” and perhaps even specify them as “somatic hallucinations, orgasmic type.”

**Table 4:** Classification of sexual hallucinations.

**Type of hallucination**	**Characterization**
Auditory sexual hallucination	Verbal or non-verbal auditory hallucination with an explicit sexual content
Coenesthetic sexual hallucination	Sexual hallucination that alters the patient's sense of physical identity (including hallucinated gender transformations)
Compound sexual hallucination	Sexual hallucination experienced in various sensory modalities simultaneously
Gustatory sexual hallucination	Gustatory hallucination that mimics tastes associated with sex
Multimodal sexual hallucination	Sexual hallucination in various sensory modalities, experienced serially
Olfactory sexual hallucination	Olfactory hallucination that mimics odors associated with sex
Somatic sexual hallucination	Sexual hallucination experienced inside the body (including orgasmic hallucinations and genital hallucinations of penetration)
Tactile sexual hallucination	Sexual hallucination experienced as sensation of touch (including genital hallucinations of touch)
Visual sexual hallucination	Visual hallucination with an explicit sexual content

### Limitations

The present study has some limitations. First, the selection of eligible patients depended on the efforts of the treating physicians and psychiatrists, rather than on a systematic screening of all patients newly admitted to the two departments by ourselves. This may have been another reason (apart from the methodological issues mentioned above) why the prevalence rate in our sample was relatively low. On the other hand, patients may have felt more comfortable discussing their sexual hallucinations with professionals whom they already knew and trusted (13), and this procedure may therefore also have been an advantage. The other way around, it may also have been a disadvantage, since not all patients referred by their treating physician or psychiatrist were considered eligible for inclusion by the interviewers, which may have been due to the fact that they trusted their own health professional more, and refused to report them to the interviewers. In any case, the connection that we established between sexual hallucinations and childhood trauma was not likely to be affected by this selection. A second limitation (basically a corollary of the first one) is that the relatively small number of cases precluded the possibility of establishing any relationship between the types of sexual hallucination and trauma. Thirdly, the present study relied on the clinical diagnoses of schizophrenia spectrum disorder and PTSD as established by the treating health professionals. Fourth and finally, recall bias may have influenced the outcome of the trauma-related questions of the CTQ, even though prior studies show similarly high trauma scores in psychotic patients, and psychotic patients have repeatedly been found to be reliable with their recall on the CTQ (7, 44). Since we did not use the denial scale of the CTQ, we have no indication whether childhood trauma may or may not have been underreported in our sample.

## Conclusions

In this clinical population of patients diagnosed with a schizophrenia spectrum disorder, we found a 1-year prevalence rate for sexual hallucinations of 0.017. All patients reported one or more types of childhood trauma, with an OR for sexual trauma of 8.7. As the differential diagnosis for sexual hallucinations also includes somatic conditions such as temporal lobe epilepsy, narcolepsy, intoxications, and PGAD, we recommend auxiliary investigations to rule out these conditions, even in the presence of a psychiatric diagnosis of schizophrenia spectrum disorder. For research purposes, we advocate further studies in transdiagnostic settings. In conformity with studies on trauma-related auditory hallucinations, we suggest additional studies to investigate the usefulness of trauma-focused therapy for sexual hallucinations. To facilitate diagnosis and research, we also recommend the use of a standardized classification of sexual hallucinations in accordance with the sensory modalities involved.

## Ethics statement

This study was carried out with written informed consent from all subjects. All subjects gave written informed consent in accordance with the Declaration of Helsinki. The protocol was approved by the Parnassia Academy of Parnassia Psychiatric Institute, The Hague, the Netherlands.

## Author contributions

JDB: contributed to the conception and design of the work, and to the analysis and interpretation of data for the work, drafted and revised the work, gave final approval for the final version to be published, and agreed to be accountable for all aspects of the work in ensuring that questions related to the accuracy or integrity of any part of the work are appropriately investigated and resolved; EM: contributed to the conception and design of the work, and to the acquisition, analysis, and interpretation of data for the work, drafted and revised the work, gave final approval for the final version to be published, and agreed to be accountable for all aspects of the work in ensuring that questions related to the accuracy or integrity of any part of the work are appropriately investigated and resolved.

### Conflict of interest statement

The authors declare that the research was conducted in the absence of any commercial or financial relationships that could be construed as a potential conflict of interest.
