# Supplementary material for: Sexual Hallucinations in Schizophrenia Spectrum Disorders and Their Relation With Childhood Trauma
Source: Front Psychiatry. 2018 May 9;9:193. doi: 10.3389/fpsyt.2018.00193 (PMC5954108; doi:10.3389/fpsyt.2018.00193)
Supplement: Supplementary file 1 [file Data_Sheet_1.PDF]

Questionnaire for Psychotic Symptoms with a Sexual Content

Translation of the Dutch questionnaire *Psychotische Symptomen met een Seksuele Inhoud*
Questionnaire compiled by Esmeralda Mangoenkarso, MD, Parnassia Psychiatric Institute, The Hague, 2012
Translation by Jan Dirk Blom, MD, PhD, Leiden University, Leiden, 2018

Patient number:
Date:

1. Demographics

Sex: M / F

Ethnicity:

Age:

Education:

Occupation:

Place of residence:

Marital status:

2. Disease-related

Clinical / Outpatient

If clinical: Date of discharge

If clinical: Reason for admission:

If clinical: Duration of admission:

If clinical: Date of admission

Duration of illness:

Current medication:

Use of illicit substances: yes/ no (if yes, which ones?)

Alcohol use:

Tobacco use:

DSM-IV diagnosis:

Axis I:

Axis II:

Axis III:

Axis IV:

Axis V:

GAF: current (…………) and past year (………….)

3. Sexual delusions and hallucinations

Symptom	Currently present	Currently absent
1. Delusion of being spied on while taking a shower		
2. Delusion of being spied on in the restroom		
3. Delusion of being spied on while undressing		
4. Delusion of being spied on otherwise, please elucidate…		
5. Sexual delusion with themes such as jealousy or infidelity of a loved one		
6. Delusion of sexual metamorphosis:<br>a. No longer being male or female<br>b. Being a neuter or eunuch<br>c. Having swapped gender; male to female or female to male		
7. Delusion regarding an alteration of the size or shape of one's genital organs		
8. Delusion of being persecuted for sexual reasons		
9. Delusion regarding sexual behaviour of others		
10. Delusion regarding one's own sexual behaviour		
11. Delusion regarding body parts and body		

movements having a sexual connotation		
12. Delusion of being pregnant		
13. Tactile hallucination of being touched improperly		
14. Tactile hallucination of being sexually abused		
15. Tactile hallucination of fluids upon the skin, with a sexual connotation		
16. Visual hallucination of the face of an ‘abuser’		
17. Visual hallucination of the body of an ‘abuser’		
18. General visual hallucination with a sexual content		
19. Auditory hallucination with a sexual content		
20. Auditory hallucination of the voice or other sounds (such as footsteps) made by an ‘abuser’		
21. Olfactory hallucination of a sexual nature		
22. Gustatory hallucination of a sexual nature		
23. Genital hallucination		
24. Erotic sensation in a different body part		
25. Improper sexual arousal		
26. Improper orgastic sensations		
27. Other psychotic symptom of a sexual nature; please elucidate…		
